# Nextclade data set for the ORF5-based lineage classification of PRRSV-1

**DOI:** 10.1128/mra.00303-25

**Published:** 2025-05-27

**Authors:** Michael Zeller, Jennifer Chang, Giovani Trevisan, Phillip C. Gauger, Jianqiang Zhang

**Affiliations:** 1 Department of Veterinary Diagnostic and Production Animal Medicine, Iowa State University 1177, Ames, Iowa, USA; 2 Vaccine and Infectious Disease Division, Fred Hutchinson Cancer Institute, Seattle, Washington, USA; Indiana University, Bloomington, Bloomington, Indiana, USA

**Keywords:** betaarterivirus, porcine reproductive and respiratory syndrome virus, PRRSV-1, *Betaarterivirus europensis*, Nextclade

## Abstract

A Nextclade data set for PRRSV-1 ORF5 based on a global nomenclature for standardized lineage classification was developed. This tool enables rapid sequence analysis, visualization, and comparison with reference strains and vaccines. By providing accessibility, it facilitates broader adoption of PRRSV-1 classification frameworks for research and surveillance.

## ANNOUNCEMENT

Porcine reproductive and respiratory syndrome virus (PRRSV) is an economically significant swine pathogen, first identified as the causative agent of Blue Ear disease in 1991–1992 (1, 2). PRRSV is divided into two species, *Betaarterivirus europensis* (PRRSV-1) and *Betaarterivirus americense* (PRRSV-2), with the Lelystad strain (GenBank M96262) serving as the prototype for PRRSV-1 and the VR-2332 strain (GenBank U87392) as the prototype for PRRSV-2. Genetic characterization of PRRSV is primarily focused on open reading frame 5 (ORF5), selected due to its high genetic diversity and the abundance of available sequences worldwide (3, 4). While multiple, progressive nomenclatures exist for PRRSV-2 ORF5 (3, 5–8), PRRSV-1 ORF5 classifications have been more limited and often regionally based (3, 9, 10). Recently, a global sequence-based nomenclature was proposed for PRRSV-1, unifying the previous classification schemes (11). To facilitate the adoption of this global nomenclature and improve usability, we have developed a Nextclade data set for PRRSV-1 ORF5 sequence classification. Nextclade is a web-based tool for rapid lineage assignment and has previously been used to establish an ORF5 data set for PRRSV-2 (12, 13).

All sequences and associated metadata were obtained from the supplemental file provided by Yim-im et al. (11). Metadata included the GenBank accession number, assigned lineage, year of collection, and country. For samples missing the year of collection, the year of GenBank submission was used instead. The Lelystad (M96262) strain was selected as the primary reference sequence due to its historical significance. Additional metadata on nucleotide and amino acid mutations relative to the reference were generated using the *augur ancestral* and *augur translate* subcommands. The data set’s phylogenetic tree was midpoint rooted, and colors were assigned for the country, year, and lineage metadata. Five PRRSV-1 vaccine sequences were included in the data set: *Porcilis, Pyrsvac, Unistrain/Amervac, PRRSVFLEX EU,* and *Suvaxyn,* each vaccine annotated as an “X” on the tree. Alignment parameters were adjusted to allow a minimum sequence length of 400 nucleotides (~65% of the sequence) and a minSeedCoverage of 0.01.

**Fig 1:**
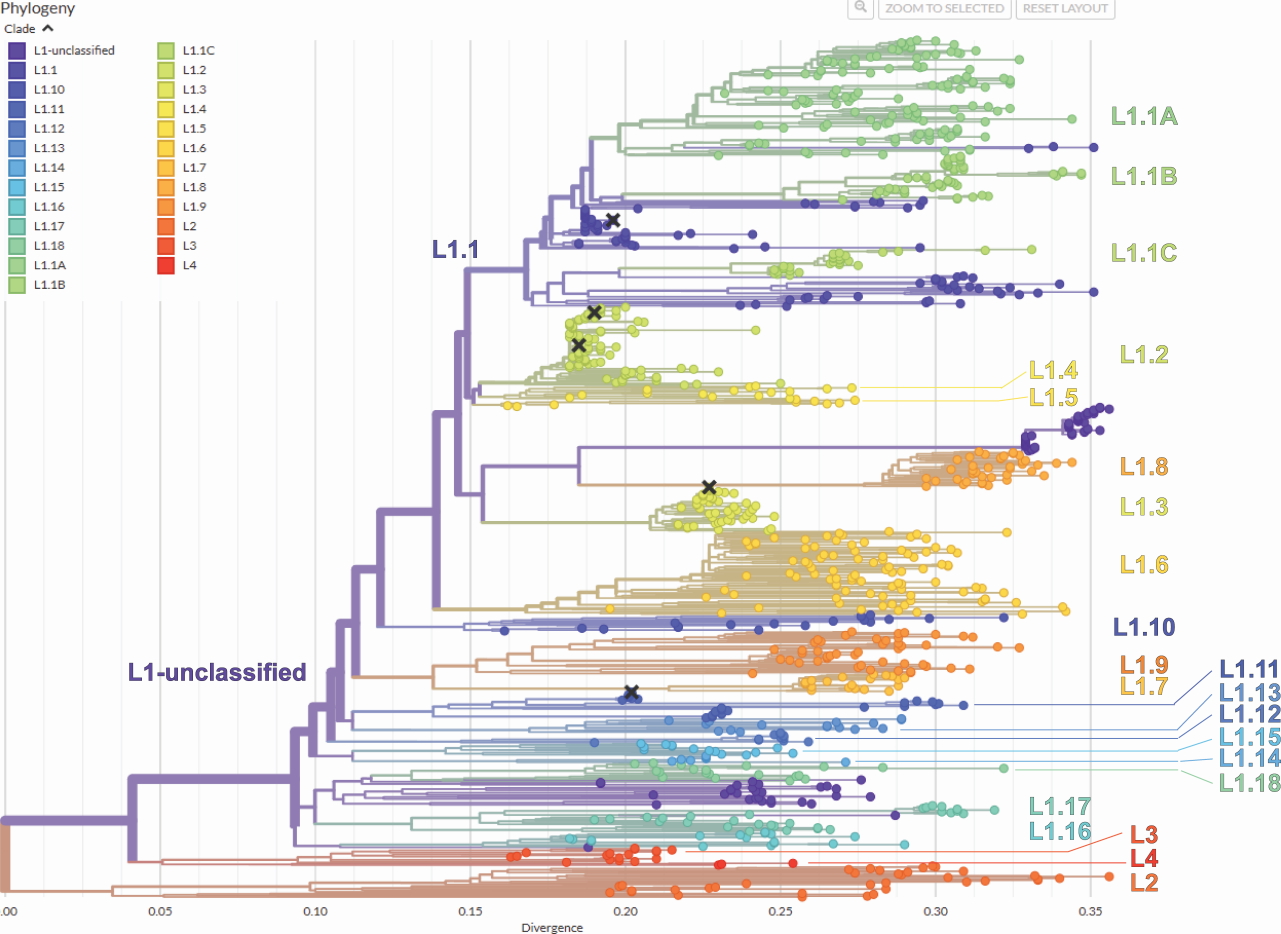
The complete tree from the PRRSV-1 Nextclade data set. The tree is colored by lineage, with additional labels added for clarity.

The final data set consisted of 967 PRRSV-1 ORF5 sequences from 23 countries. The use of Nextclade provides standardization of the classification workflow, by combining the reference set from (11) with the built-in methodology. To use this tool, users simply upload their sequences (Fig. 1). Upon submission, Nextclade generates a table displaying the inferred lineage, sequence quality metrics, and mutations relative to the reference. A secondary screen visualizes the placement of user-submitted sequences on a neighbor-joining tree, allowing for comparison with other sequences, including vaccines. All results are available for direct download to facilitate storage and further analysis. Nextclade provides a user-friendly platform for accurate PRRSV lineage assignment, supporting the adoption of the new PRRSV nomenclature.

## Data Availability

This web resource is accessible from Nextclade directly (https://clades.nextstrain.org/) or from ISU PRRSView (http://prrsv.vdl.iastate.edu). The data sets generated for this study can be directly accessed in the Nextclade GitHub repository (https://github.com/nextstrain/nextclade_data/tree/master/data/community/isuvdl/mazeller/prrsv1/orf5/yimim2025).
